# Microarray-based estimation of SNP allele-frequency in pooled DNA using the Langmuir kinetic model

**DOI:** 10.1186/1471-2164-9-605

**Published:** 2008-12-16

**Authors:** Bin-Cheng Yin, Honghua Li, Bang-Ce Ye

**Affiliations:** 1 Laboratory of Biosystems and Microanalysis, State Key Laboratory of Bioreactor Engineering, East China University of Science & Technology, Shanghai, 200237, PR China; 2 Department of Molecular Genetics, Microbiology and Immunology, University of Medicine and Dentistry of New Jersey-Robert Wood Johnson Medical School, SRB 110, 661 Hoes Lane, Piscataway, NJ 08854, USA

## Abstract

**Background:**

High throughput genotyping of single nucleotide polymorphisms (SNPs) for genome-wide association requires technologies for generating millions of genotypes with relative ease but also at a reasonable cost and with high accuracy. In this work, we have developed a theoretical approach to estimate allele frequency in pooled DNA samples, based on the physical principles of DNA immobilization and hybridization on solid surface using the Langmuir kinetic model and quantitative analysis of the allelic signals.

**Results:**

This method can successfully distinguish allele frequencies differing by 0.01 in the actual pool of clinical samples, and detect alleles with a frequency as low as 2%. The accuracy of measuring known allele frequencies is very high, with the strength of correlation between measured and actual frequencies having an r^2 ^= 0.9992. These results demonstrated that this method could allow the accurate estimation of absolute allele frequencies in pooled samples of DNA in a feasible and inexpensive way.

**Conclusion:**

We conclude that this novel strategy for quantitative analysis of the ratio of SNP allelic sequences in DNA pools is an inexpensive and feasible alternative for detecting polymorphic differences in candidate gene association studies and genome-wide linkage disequilibrium scans.

## Background

Single nucleotide polymorphisms (SNPs) represent the most genetic variation in the human genome, and are thought to have a promising future in a wide range of human genetics applications such as pharmacogenomics, population evolution, functional genomics, forensic and identification of genes responsible for the susceptibility of complex diseases. It has been suggested that 30,000–500,000 SNPs are required for a whole-genome association study [1,2]. Accurate determination of allele frequencies of such a large number of SNPs in a large number of human samples is an unusual challenge in the whole genome association studies for genetic alterations of low relative risk [3]. It not only involves heavy workload, unusual amount of time and cost, but also a large amount of DNA of each sample. Because only very few markers are expected to show linkage and/or association in family data, a simple, highly efficient and cost-effective screening approach to identification of genetic markers showing linkage and/or association is highly desirable. Using pooled DNA samples may significantly facilitate meeting this goal since hundreds of DNA samples can be reduced to a single sample. Although pooling DNA samples may result in a loss of information of haplotype information, it is still appealing because of the tradeoff of the significant reduction in the amounts of effort and cost.

A number of approaches used for SNP genotyping have been used to estimate allele frequency in pooled DNA samples. These include primer extension followed by DHPLC [4], allele-specific amplification with real-time PCR [5], BAMPER [6], TAQMAN™ and RFLP analysis [7], dynamic allele-specific hybridization (DASH) [8], MassARRAY™ [9], mass spectrometry [10], pyrosequencing [11,12], SSCP [13], the amplification refractory mutation system (ARMS) [14], and DNA microarrays [15,16]. However, most of these methods are based on substantial post-PCR processing, and for one or very few SNPs for one pooling sample as a time. In this report, we describe a new microarray-based method for estimating the allele frequency in pooled DNA samples based on the physical principles of DNAs immobilization and hybridization on solid surface. This method well suits large-scale genetic association study, and has a number of advantages: capability of scaling up both in the numbers of SNPs and pooled samples (cases and controls) by utilizing microarray platform, assay of thousands of SNPs on one chip under uniform conditions, employing only two universal fluorescently labeled tags for thousands of SNPs, and no post-PCR processing.

The physical principle of hybridization on chip surface, modeled as a Langmuir adsorption process, has been extensively studied in recent years. However, most studies concentrate on the kinetics and thermodynamics of hybridization for gene expression assay [17,18] and genotyping [19]. To our knowledge, the present study is the first application of the Langmuir function to SNP allele-frequencies estimation using pooled DNA and microarray.

Six SNP markers, two, *ESR1E-U11 *(T/C, rs11155816) and *ESR1F-U21 *(A/G, rs9340799) in the *ESR1 *gene, one, *TGFB1D-U2 *(G/C, rs1800471) in the *TGFB1 *gene, and three, *HBB17 *(A/T, c.102 A > T), *HBB28 *(T/C, rs33931746) and *HBB26 *(C/T, rs33950507) in the *HBB *gene, were employed to demonstrate the feasibility of this approach. The estrogen receptor encoded by *ESR1*, is a ligand-activated transcription factor composed of several domain important for hormone binding, DNA binding and activation of transcription. It has widely been demonstrated that *ESR1 *polymorphism is associated with breast cancer and bone mineral density. TGFB is a multifunctional peptide that controls proliferation, differentiation, and other functions in many cell types. It is well known that TGFB plays an important role in human diseases. Mutations in the TGF-beta pathway are responsible for many biological processes in cancer development. The increased expression and a polymorphism of *TGFB1 *have also been associated with abdominal obesity in humans. The inherited blood disorder β-thalassemia is caused by mutations in the *HBB *gene, which markedly decreases or completely prevents the production of β-globin chains. It is the most common inherited single-gene disorders in the world with the highest prevalence in many areas of southern China including Taiwan, and has been and/or remains endemic.

## Results

### Principle and Design of the Method

**Figure 1:**
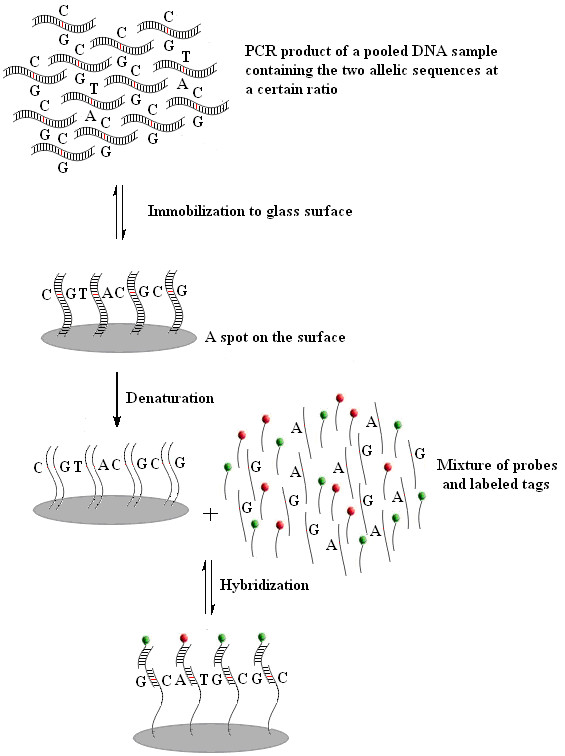
**Schematic illustration of the competitive immobilization and hybridization processes on glass surface using the strategy of tag-probe-allele sandwich structure**. The single-base variation is indicted by letters. Fluorescent dyes are represented by small red or green spheres.

Six SNP markers, two, *ESR1E-U11 *(T/C, rs11155816) and *ESR1F-U21 *(A/G, rs9340799) in the *ESR1 *gene, one, *TGFB1D-U2 *(G/C, rs1800471) in the *TGFB1 *gene, and three, *HBB17 *(A/T, c.102 A > T), *HBB28 *(T/C, rs33931746), and *HBB26 *(C/T, rs33950507) in the *HBB *gene, were included to demonstrate the feasibility of our new approach. When the PCR product from a pooled DNA sample containing the two alleles at a given ratio for an SNP is spotted onto the glass slide surface, competition between the two allelic sequences may occur during surface immobilization (Figure 1) because the spot has limited binding capacity determined by the active groups and steric hindrance. As a result, the amounts of the two allelic sequences bound to the spot should be proportional to the initial amounts in the PCR product. When two fluorescently labeled allele-specific probes hybridize to their corresponding allelic sequences in the spot, the intensities of the two fluorescent colors in the spot should reflect the relative amounts of allelic sequences in the pooled DNA sample.

Two allele-specific probes were designed for each SNP. Each probe was composed of a specific domain (~15 bp) to hybridize with its allelic sequences at their 5'-ends, and a 3'-segment consisting of one of two 20-bp universal sequences to hybridize with a fluorescence-labeled universal tag. The two universal tags (Cy5-TTACGTGATGGTAATAGTGCTG and Cy3-TTAGGTCGTAGGTGCGTTAGAT) labeled with different fluorescent dyes, were designed to perfectly match their corresponding universal sequences of the SNP probes to form a "sandwich" architecture with the PCR product. The two universal tags were used for all SNPs under analysis. The sequences of the probes and the universal tags were carefully selected to avoid formation of tag-to-tag hybridization and hair-pin structures, which may interfere with the hybridization between tags and their probes, and between probes and their allelic sequences [20]. The strategy of tag-probe-alleles sandwich complex is compatible with high-throughput analysis, flexible in experimental design, and cost effective.

Based on the analysis of the thermodynamics of immobilization and hybridization process on the solid surface, we constructed the Langmuir-type isotherm model to integrate the two processes. The Langmuir-type parameters of the model were obtained from a series of allele frequency experiment. Several theoretical considerations and experimental constraints were imposed as follows: (a) the immobilization process was viewed as a reversible competitive bimolecular surface reaction (adsorption and desorption) between DNA molecules and the glass surface; (b) the immobilization was allowed to reach equilibrium under the defined experimental conditions; and (c) all PCR products were diluted to the same concentration before printed onto the slides. Under these conditions, we can deduce the equations to characterize the relationship between the concentrations $c_{A}^{\left(i\right)}$ and $c_{B}^{\left(i\right)}$ of the allele sequences, *A *and *B*, immobilized to the glass surface and the initial concentrations of these alleles in the spotted solution as follows [21,22],

(1) $$\frac{\partial c_{A}^{\left(i\right)}}{\partial t}=k_{f,A}^{\left(i\right)}(R-c_{A}^{\left(i\right)}-c_{B}^{\left(i\right)})c_{A}-k_{r,A}^{\left(i\right)}c_{A}^{\left(i\right)},$$

(2) $$\frac{\partial c_{B}^{\left(i\right)}}{\partial t}=k_{f,B}^{\left(i\right)}(R-c_{A}^{\left(i\right)}-c_{B}^{\left(i\right)})c_{A}-k_{r,B}^{\left(i\right)}c_{B}^{\left(i\right)},$$

where the superscripted "*i*" represents the surface immobilization values, $k_{f,A}^{\left(i\right)}$, $k_{r,A}^{\left(i\right)}$, $k_{f,B}^{\left(i\right)}$ and $k_{r,B}^{\left(i\right)}$ represent the rate constants of immobilization association and dissociation of alleles *A *and *B*, respectively, *R *is the maximum surface concentration of the active group on a spot, *c*_*A *_and *c*_*B *_represent the spotting concentrations of alleles *A *and *B *in the pooled DNA sample, $c_{A}^{\left(i\right)}$ and $c_{B}^{\left(i\right)}$ represent the surface concentrations of the immobilized allelic sequences *A *and *B*.

At the equilibrium $\partial c_{A}^{\left(i\right)}/\partial t=0$, $\partial c_{B}^{\left(i\right)}/\partial t=0$, the dynamic equilibrium of *Eqs*. (1) and (2) can be transformed to deduce the immobilized allele concentration as follows (the suffix "*eq*" stands value at equilibrium),

(3) $$c_{eq,A}^{\left(i\right)}=\frac{RK_{f,A}^{\left(i\right)}c_{A}k_{r,B}^{\left(i\right)}}{k_{f,A}^{\left(i\right)}c_{A}k_{r,B}^{\left(i\right)}+k_{f,B}^{\left(i\right)}c_{B}k_{r,A}^{\left(i\right)}+k_{r,A}^{\left(i\right)}k_{r,B}^{\left(i\right)}},$$

(4) $$c_{eq,B}^{\left(i\right)}=\frac{RK_{f,B}^{\left(i\right)}c_{B}k_{r,A}^{\left(i\right)}}{k_{f,A}^{\left(i\right)}c_{A}k_{r,B}^{\left(i\right)}+k_{f,B}^{\left(i\right)}c_{B}k_{r,A}^{\left(i\right)}+k_{r,A}^{\left(i\right)}k_{r,B}^{\left(i\right)}},$$

Let $K_{a,A}^{\left(i\right)}=\frac{k_{f,A}^{\left(i\right)}}{k_{r,A}^{\left(i\right)}}$ and $K_{a,B}^{\left(i\right)}=\frac{k_{f,B}^{\left(i\right)}}{k_{r,B}^{\left(i\right)}}$ represent immobilization equilibrium constants, then we can rewrite *Eqs*. (3) and (4) as follows,

(5) $$c_{eq,A}^{\left(i\right)}=R\frac{K_{a,A}^{\left(i\right)}c_{A}}{1+K_{a,A}^{\left(i\right)}c_{A}+K_{a,B}^{\left(i\right)}c_{B}}=\alpha K_{a,A}^{\left(i\right)}c_{A},$$

(6) $$c_{eq,B}^{\left(i\right)}=R\frac{K_{a,B}^{\left(i\right)}c_{B}}{1+K_{a,A}^{\left(i\right)}c_{A}+K_{a,B}^{\left(i\right)}c_{B}}=\alpha K_{a,B}^{\left(i\right)}c_{B},$$

where the factor *α *is defined as,

(7) $$\alpha =\frac{R}{1+K_{a,A}^{\left(i\right)}c_{A}+K_{a,B}^{\left(i\right)}c_{B}}.$$

Because the two alleles of each SNP only differ by one base, and the immobilization conditions and the chemical properties of glass surface are the same to these two alleles, in theory, the values of the immobilization equilibrium constants $K_{a,A}^{\left(i\right)}$ is similar to $K_{a,B}^{\left(i\right)}$, ($K_{a,A}^{\left(i\right)}\approx K_{a,B}^{\left(i\right)}$) to certain extent. During the experiment, all PCR products of the pooled DNA samples were diluted to the same concentration for spotting, *c*_*R*_(*c*_*R *_= *c*_*A *_+ *c*_*B*_), which means *c*_*R *_is a constant in this assay. Then *Eq*. (7) can be rewritten as,

(8) $$\alpha =\frac{R}{1+K_{a,A}^{\left(i\right)}c_{R}}.$$

On the basis of *Eq*. (8), *α *value only depends on the slide surface chemistry (*R*), immobilization reaction rate constants ($K_{a,A}^{\left(i\right)}$ and $K_{a,B}^{\left(i\right)}$), and the allelic sequence concentrations in the spotted solution (*c*_*A *_and *c*_*B*_). Thus, α can be considered as a constant under the defined experimental conditions.

Generally, the hybridization kinetics on the solid surface is represented by the familiar relationship,

(9) $$allele+probe-tag\overset{k_{a}}{\underset{k_{d}}{⇄}}allele-probe-tag$$

It is well known that the simplest model for DNA hybridization on a chip is the Langmuir kinetic model for adsorption [23,24]. Langmuir adsorption theory for microarray is based on the assumption that there are two competing processes: the adsorption process, which is binding the probe molecules to the immobilized DNA molecules to form duplexes, and desorption, which is the reverse process of duplexes dissociating into separate molecules. Thus the form of the equation results in a non-linear relation between the concentrations and the signal intensities. In our method, some theoretical considerations and experimental constraints are imposed to fit the conditions required by the Langmuir regime: (a) the labeled tag-probe is in large excess compared to allelic sequences immobilized on the surface; (b) the tag-probe concentration near the slide surface is assumed to be constant and equal to the bulk concentration during hybridization process;.(c) the hybridization reaction can achieve equilibrium.

If we neglect some effects such as that secondary structures and cross-hybridization, and there is only specific binding between a given probe and its complimentary allelic strand, and between labeled tag and its corresponding probe, we can deduce that the amount of labeled tag-probe bound to the complementary immobilized allelic sequence is proportional to the fluorescence intensity. Then the two fluorescence intensities on the same spot could be translated into the relative amounts of the two alleles in the pooled DNA sample. By recasting the result to yield the background-corrected intensity, *I_A_* and *I_B_*, as a function of a series of allele immobilization concentrations, $c_{A}^{\left(i\right)}$ and $c_{B}^{\left(i\right)}$, we obtain the following equation (the superscripted "*h*" means the hybridization fluorescence values),

(10) $$I_{A}=I_{R,A}\frac{K_{a,A}^{\left(h\right)}c_{A}^{\left(i\right)}}{1+K_{a,A}^{\left(h\right)}c_{A}^{\left(i\right)}},$$

(11) $$I_{B}=I_{R,B}\frac{K_{a,B}^{\left(h\right)}c_{B}^{\left(i\right)}}{1+K_{a,B}^{\left(h\right)}c_{B}^{\left(i\right)}}.$$

where $K_{a,A}^{\left(h\right)}$ and $K_{a,B}^{\left(h\right)}$ represent hybridization equilibrium constants, the factor *I*_*R*,*A *_and *I*_*R*,*B *_refer to the maximal values related to the total number of molecules of the alleles immobilized in the given spot, and the intensities *I*_*A *_and *I*_*B *_refer to the background-corrected intensities of alleles *A *and *B *from a given spot.

Combining with the *Eqs*. (5) and (6), we can rewrite the *Eqs*. (10) and (11) as follows,

(12) $$I_{A}=I_{R,A}\frac{K_{a,A}^{\left(h\right)}(\alpha K_{a,A}^{\left(i\right)}c_{A})}{1+K_{a,A}^{\left(h\right)}(\alpha K_{a,A}^{\left(i\right)}c_{A})}=I_{R,A}\frac{K_{A}c_{A}}{1+K_{A}c_{A}},$$

(13) $$I_{B}=I_{R,B}\frac{K_{a,B}^{\left(h\right)}(\alpha K_{a,B}^{\left(i\right)}c_{B})}{1+K_{a,B}^{\left(h\right)}(\alpha K_{a,B}^{\left(i\right)}c_{B})}=I_{R,B}\frac{K_{B}c_{B}}{1+K_{B}c_{B}}.$$

where the factor *K*_*A *_and *K*_*B *_are defined as $K_{A}=\alpha K_{a,A}^{\left(h\right)}K_{a,A}^{\left(i\right)}$, and $K_{B}=\alpha K_{a,B}^{\left(h\right)}K_{a,B}^{\left(i\right)}$. The relationship between the fluorescence intensity of binding sandwich and allele spotting concentration approximately follows the Langmuir-type adsorption isotherm function (*Eqs*. 12 and 13). This observed Langmuir-type isotherm model integrates the thermodynamics of the immobilization and hybridization processes.

Assuming that the allele concentration ratio (*c*_*A*_/*c*_*B*_) remains constant in the spotting solution and the initial pooled DNA sample, which means the alleles are equally amplified. By transforming and combining *Eqs*. (12) and (13), we can determine the pool allele frequency by the following equations,

(14) $$f_{A}=\frac{c_{A}}{c_{A}+c_{B}}=\frac{I_{A}(I_{R,B}-I_{B})K_{B}}{I_{A}(I_{R,B}-I_{B})K_{B}+I_{B}(I_{R,A}-I_{A})K_{A}},$$

(15) *f*_*B *_= 1 - *f*_*A*_.

where *f*_*A *_and *f*_*B *_represent the alleles *A *and *B *frequencies in the pooled DNA sample.

### Characterization of the kinetics of microarray

**Figure 2:**
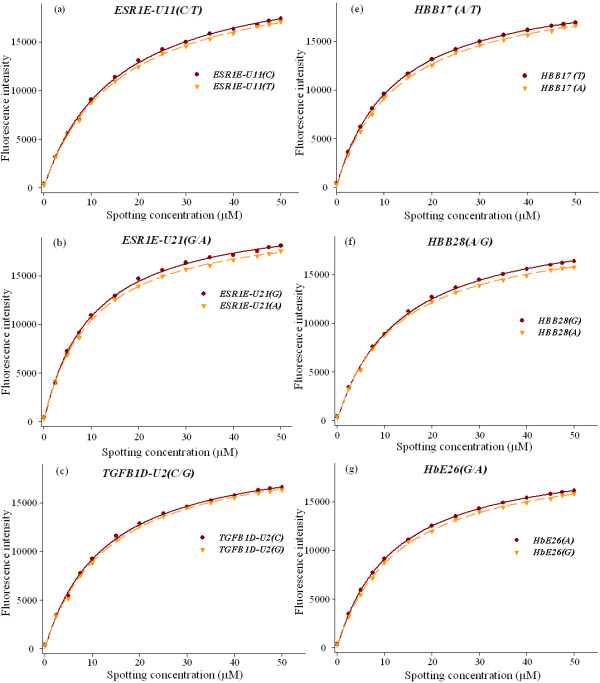
**Average background-corrected signal intensity as a function of allele spotting concentration for the six SNPs**. All curves depict the non-linear relationship modeled as Langmuir kinetic function for signal intensity from microarray. The spotting concentration of the allelic sequences ranged from 0 to 50 μM for each SNP. The signal intensity is calculated by integrating line profiles for spots containing the perfect match and normalizing the signals. The solid and dashed lines in each plot show best fit to the Langmuir kinetic model for the two alleles.

In order to determine the values of *K*_*a *_(*K*_*A *_or *K*_*B*_) and *I*_*R*_, a series of pooled reference DNA samples with different ratios between the two allelic sequences of each of the six SNPs at different proportions for the six SNPs (*ESR1E-U11(T/C), ESR1F-U21(A/G), TGFB1D-U2(G/C)*, *HBB17(A/T), HBB28(T/C)*, and *HBB26(C/T)*) were prepared from two DNA samples homozygous for the two alleles (see Method). For each SNP, fourteen pooled reference DNA samples were prepared with allele ratios ranging from 0% to 100%. The samples were amplified by PCR, purified, brought to a final total concentration of 50 μM, and printed onto a glass slide. Each sample was spotted five times for evaluating spot-to-spot variation. The fabricated array was then incubated in a hybridization solution containing Cy3/Cy5 labeled tag-probe duplexes which were in large excess over the number of immobilized allelic molecules on the array. The plots of the allele concentrations in spotting solution versus average background-corrected fluorescence intensities for the six SNPs were analyzed using the Langmuir-type model (*Eqs*. 12 and 13). Results are shown in Figure 2.

The values of constants, *K*_*a *_and *I*_*R*_, for the six SNPs derived from the best-fit Langmuir isotherms are presented in Table 1. *I*_*R *_is the maximum fluorescence intensity, representing the saturated hybridization signal for the immobilized allele. In general, *I*_*R *_is determined by hybridization conditions, immobilization conditions, and surface chemistry of the slide, but independent from the spotting concentration if the amount of allelic sequences in the spotting solution is in a great excess over the absorbance capacity of the slide surface. In *Eqs*. (12) and (13), *K*_*a *_is the observed apparent equilibrium constant of reaction between the probes in hybridization solution and the allele DNA in spotting solution. It is determined by immobilization affinity (*K*_*a *_^(*i*)^), hybridization affinity (*K*_*a *_^(*h*)^), surface chemistry (*R*, surface concentration of active group), and the total spotting concentration (*c*_*R*_). As shown in Table 1, the observed equilibrium constant *K*_*a *_has the same order of magnitude for six SNPs: 0.65 × 10^5 ^M^-1^, 0.66 × 10^5 ^M^-1 ^for genotype *T *and *C *of *ESR1E-U11*; 0.99 × 10^5 ^M^-1^, 0.99 × 10^5 ^M^-1 ^for genotype *A *and *G *of *ESR1F-U21*; 0.79 × 10^5 ^M^-1^, 0.80 × 10^5 ^M^-1 ^for genotype *G *and *C *of *TGFB1D-U2*, 0.51 × 10^5 ^M^-1^, 0.52 × 10^5 ^M^-1 ^for genotype *A *and *T *of *HBB17*; 0.77 × 10^5 ^M^-1^, 0.79 × 10^5 ^M^-1 ^for genotype *A *and *G *of *HBB28*; 0.79 × 10^5 ^M^-1^, 0.80 × 10^5 ^M^-1 ^for genotype *G *and *A *of *HBB26 *respectively. These results clearly demonstrated that the single-base difference of the PCR products of each SNP had negligible effect on immobilization and hybridization for the perfect-matched probes.

**Table 1:** The constants of Ka and IR for tag-probe-allele hybridization derived from spike-in experiments

SNP	Allele	*K*_*a *_(×10^6^/M^-1^)	*I*_*R *_(×10^4^)
*ESR1E-U11*	*T*	6.5	2.230
	*C*	6.6	2.268

*ESR1F-U21*	*A*	9.9	2.092
	*G*	9.9	2.170

*TGFB1D-U2*	*G*	7.9	2.082
	*C*	8.0	2.083

*HBB17(A)*	*A*	5.1	2.082
	*T*	5.2	2.099

*HBB28(A)*	*A*	7.7	1.979
	*G*	7.9	2.082

*HBB26(G)*	*G*	7.9	1.983
	*A*	8.0	1.999

### Allele frequency estimation for pooled DNA samples from synthetic DNAs and plasmids

Since experimental factors such as immobilization condition, hybridization, and surface chemistry may affect the values of *K*_*a *_and *I*_*R*_, it is better to include the reference samples with known allele frequencies as controls in each sub-array. With the values of *K*_*a *_and *I*_*R *_derived from the experimental data, it is possible to estimate the allele frequencies in the pooled samples. This was demonstrated by using pooled DNA samples with different ratios (frequency from 5% to 100%) between the two allelic sequences, prepared by mixing two DNA samples homologous from either allele from synthetic or plasmid DNA stocks. The allele frequencies of all samples were calculated using *Eqs*. (14) and (15). As summarized in Table 2, the mean standard deviations (SD) between the five replicates for the six SNPs were from 0.23 to 0.26 with a maximal range of 0.011 to 0.041 for each SD. These results confirm the reliability of our method.

**Table 2:** Allele frequency estimates for the six SNPs from pooled DNA samples*

			Allele Frequency											
			
SNP	Allele	Measure	Poo1 1	Poo1 2	Poo1 3	Poo1 4	Poo1 5	Poo1 6	Poo1 7	Poo1 8	Poo1 9	Poo1 10	Poo1 11	Pool 12
*ESR1E-U11*	*T*	Actual	0.05	0.10	0.20	0.30	0.40	0.50	0.60	0.70	0.80	0.90	0.95	1.00
		Observed	0.053	0.108	0.19	0.308	0.396	0.514	0.604	0.704	0.804	0.894	0.949	0.988
		SD	0.014	0.017	0.039	0.035	0.025	0.029	0.023	0.031	0.03	0.025	0.016	0.014

*ESR1F-U21*	*A*	Actual	0.05	0.10	0.20	0.30	0.40	0.50	0.60	0.70	0.80	0.90	0.95	1.00
		Observed	0.051	0.113	0.195	0.307	0.395	0.509	0.599	0.708	0.799	0.906	0.943	0.992
		SD	0.017	0.03	0.036	0.031	0.026	0.023	0.022	0.023	0.041	0.02	0.021	0.011

*TGFB1D-U2*	*G*	Actual	0.05	0.10	0.20	0.30	0.40	0.50	0.60	0.70	0.80	0.90	0.95	1.00
		Observed	0.057	0.107	0.2	0.307	0.405	0.503	0.603	0.706	0.804	0.893	0.951	0.99
		SD	0.011	0.02	0.033	0.034	0.03	0.028	0.025	0.027	0.03	0.026	0.014	0.014

*HBB17*	*A*	Actual	0.05	0.10	0.20	0.30	0.40	0.50	0.60	0.70	0.80	0.90	0.95	1.00
		Observed	0.057	0.107	0.208	0.319	0.392	0.499	0.616	0.706	0.815	0.884	0.95	0.988
		SD	0.011	0.021	0.031	0.034	0.031	0.027	0.031	0.027	0.032	0.031	0.017	0.013

*HBB28*	*A*	Actual	0.05	0.10	0.20	0.30	0.40	0.50	0.60	0.70	0.80	0.90	0.95	1.00
		Observed	0.056	0.108	0.208	0.322	0.407	0.504	0.612	0.71	0.815	0.908	0.951	0.99
		SD	0.015	0.022	0.031	0.032	0.035	0.018	0.025	0.028	0.032	0.025	0.02	0.01
*HBB26*	*G*	Actual	0.05	0.10	0.20	0.30	0.40	0.50	0.60	0.70	0.80	0.90	0.95	1.00
		Observed	0.06	0.103	0.211	0.309	0.401	0.508	0.609	0.71	0.814	0.908	0.95	0.987
		SD	0.012	0.02	0.023	0.026	0.029	0.015	0.021	0.028	0.031	0.025	0.023	0.017

*Each pooled DNA sample was measured five times.

### Allele frequency estimation of pooled genomic DNA samples

We also investigated the validity of our method for pooled genomic DNA samples from clinical specimens using the same protocols described above for the synthetic and plasmid DNA stocks. Ten genomic DNA samples with different ratios between allelic sequences were prepared by pooling 100 individual genomic DNA samples of known genotypes for SNP *HBB28(T/C)*. The fraction of the minor allele "C" ranged from 2% to 10%, with 1% increments. Twenty replicas of each pooled sample were measured to test the repeatability of the method. The variations are expressed as ± SD and ± standard error of the mean (SEM). Results are listed in Table 3. As shown, SDs ranged from 0.005 to 0.010, and SEM ranged from 0.0044 to 0.0103, indicating that our method is highly reproducible.

**Table 3:** Allele frequency estimates from pooled DNA samples with the minor allele frequency ranging from 2% to 10% for the "C" allele of SNP *HBB28*

Measurement No.	Pool 1	Pool 2	Pool 3	Pool 4	Pool 5	Pool 6	Pool 7	Pool 8	Pool 9
1	0.014	0.035	0.047	0.048	0.058	0.078	0.091	0.079	0.110
2	0.015	0.028	0.038	0.049	0.059	0.051	0.085	0.091	0.095
3	0.030	0.033	0.043	0.054	0.059	0.072	0.088	0.087	0.099
4	0.018	0.040	0.042	0.049	0.067	0.070	0.077	0.092	0.118
5	0.031	0.032	0.043	0.048	0.063	0.065	0.083	0.082	0.095
6	0.022	0.035	0.037	0.052	0.054	0.053	0.077	0.096	0.097
7	0.024	0.034	0.039	0.052	0.072	0.073	0.087	0.085	0.096
8	0.032	0.037	0.041	0.059	0.067	0.067	0.089	0.091	0.125
9	0.032	0.029	0.046	0.048	0.070	0.078	0.078	0.079	0.119
10	0.019	0.028	0.036	0.057	0.059	0.081	0.061	0.087	0.120
11	0.018	0.038	0.044	0.034	0.056	0.074	0.084	0.087	0.114
12	0.031	0.030	0.041	0.059	0.059	0.069	0.084	0.084	0.098
13	0.022	0.029	0.048	0.067	0.054	0.067	0.079	0.088	0.097
14	0.024	0.031	0.032	0.063	0.065	0.067	0.087	0.087	0.095
15	0.032	0.022	0.042	0.054	0.067	0.065	0.080	0.089	0.114
16	0.023	0.024	0.039	0.054	0.062	0.057	0.077	0.093	0.111
17	0.024	0.032	0.048	0.067	0.062	0.069	0.080	0.094	0.094
18	0.030	0.032	0.040	0.052	0.058	0.067	0.082	0.093	0.097
19	0.024	0.037	0.032	0.054	0.058	0.067	0.077	0.091	0.096
20	0.029	0.039	0.035	0.049	0.072	0.074	0.082	0.091	0.100
Actual	0.020	0.030	0.040	0.050	0.060	0.070	0.080	0.090	0.100
Observed	0.025	0.032	0.041	0.053	0.062	0.068	0.081	0.088	0.105
SD	0.006	0.005	0.005	0.007	0.006	0.008	0.006	0.005	0.010
SEM	0.0060	0.0050	0.0049	0.0077	0.0059	0.0083	0.0064	0.0044	0.0103

### Robustness of the method

In an association studies, it is critical to learn which markers with their allele frequencies significantly different among populations. The simplest strategy is to compare between results from the pooled samples of all cases and those from pooled samples of all controls. In more complex design, creating sets of sub-pools allows stratification, not only on the basis of the disease trait but also on secondary and tertiary traits as well. In these cases, it is very important to detect minor differences in allele frequencies between pools or sub-pools. To evaluate sensitivity of our method, the significance level of the differences in the allele frequencies between the two pooled samples with the closest allele frequencies (Table 3) was assessed by the Kolmogorov-Smirnov test. The resultant *P *values are listed in Table 4. As shown, samples with a difference in their allele frequencies as small as 1% could be discriminated (*P *< 0.0078) if the sampling error is negligible. Therefore, our method provides a very powerful tool for association studies using pooled samples.

**Table 4:** *P *values calculated by the Kolmogorov-Smirnov test to assess the significance levels of the differences in allele frequencies between pooled samples with closest allelic frequencies

Pools		*P *value
Pool 1	Pool 2	0.0078
Pool 2	Pool 3	0.0006
Pool 3	Pool 4	< 0.0001
Pool 4	Pool 5	0.0006
Pool 5	Pool 6	0.0078
Pool 6	Pool 7	< 0.0001
Pool 7	Pool 8	0.0078
Pool 8	Pool 9	< 0.0001

**Figure 3:**
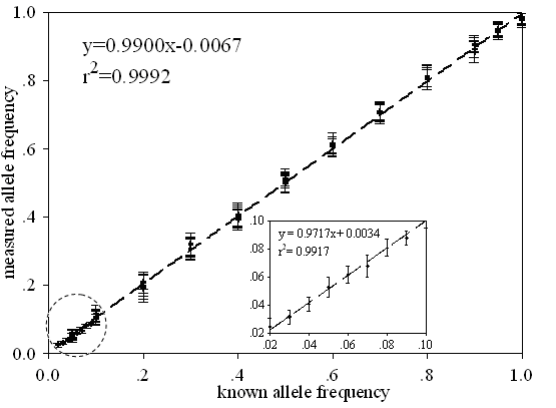
**Scatter plot demonstrating the accuracy of allele frequency estimation using pooled DNA samples**. The chart is drawn based on the results for allele frequency listed in Table 2 and Table 3 versus the known frequencies prior to PCR amplification for the six SNPs. Short horizontal bars are error bars. The diagonal line shows complete concordance between known and observed allelic fractions.

Figure 3 is a scatter plot that summarizes all allele frequency estimation data (Table 2 and Table 3) for the six markers. The estimated pooled allele frequencies are in good agreement with the results of individual genotyping. For the genomic DNA samples (Table 3), the estimates and known values of allele frequencies are highly correlated (*r*^2 ^= 0.9917). For all assays in the present study, the known and the measured allele frequencies were highly correlated with a correlation coefficient of 0.9992 (*P *< 0.01). The mean value of the differences between known and measured frequencies was 0.007. These results indicate that our method is highly accurate and reproducible. According to the data shown in Table 3, it is possible to apply our method to determining allele frequencies ≥ 2%.

**Figure 4:**
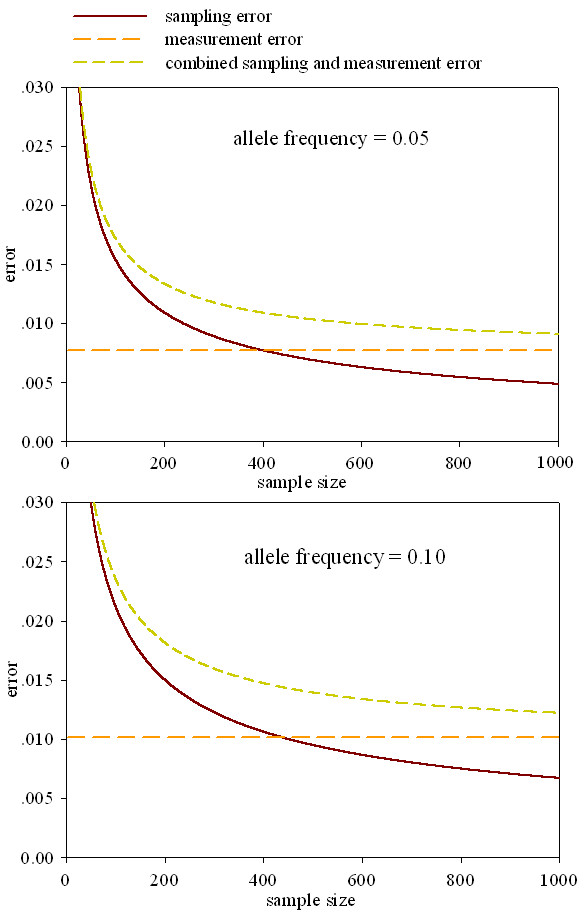
**Comparison of expected sampling errors and experimental errors of the frequencies for the two alleles of SNP *HBB28***. The chart was drawn based on the results in Table 3. (a) The solid line is the expected sampling error for SNP site (*HBB28*) for the allele frequency of 0.05 for sample size up to 1000. The upper broken line is the estimated combine sampling and measurement error for this method based on Table 3 (see text). The lower broken line is measurement error. (b) The same as (a) for the allele frequency of 0.10 described in Table 3.

To obtain a high degree of accuracy and sensitivity, several factors need to be taken into consideration. These include the number of samples to pool, the volumes of DNA to transfer by pipetting, microarray preparation and hybridization. For this reason, the reproducibility for the quantification of this method was evaluated. We investigated the sampling and measurement errors [5] using samples with allele frequencies of 0.05 and 0.10 for *HBB28(C)*. Each sample was repeated 20 times which were subdivided into four groups, five each. Then we calculated the measurement error using the standard error of the mean

$$\sigma_{m}=\sqrt{\frac{\text{S}\text{.D of measurements}}{\text{no}\text{. of measurements}}},$$

where the number of measurements was five, the measurement error is independent of sample size. The expected sampling error can be expressed as $\sigma_{s}=\sqrt{\frac{f(1-f)}{2\text{n}}}$ (*f *= allele frequency and n = sample size) [25], and the equation of $\sigma =\sqrt{\sigma_{m}^{2}+\sigma_{s}^{2}}$ is for the estimated combined sampling and measurement error. Results for the two allele frequencies are shown in Figure 4. With an actual allele frequency of 0.05, the measurement error was ± 0.0077 (Panel (a), Figure 4). For an actual allele frequency of 0.10, the measurement error was ± 0.0103 (Panel (b), Figure 4). Results in Figure 1 show that when the sample size is smaller than 400, the sampling error is greater than the measurement error at allele frequency of 0.05; when the sample size is smaller than 436, the sampling error is greater than the measurement error at allele frequency of 0.10. Thus the measurement error will be dominant in allele frequency estimation for a large sample size such as n > 500. Generally, the measurement error would be smaller than the statistical error of sampling at a large sample size. To obtain a reliable allele frequency, the sample size ≥ 500 is necessary for an allele frequencies greater than 0.10.

## Discussion

Identification of genetic loci associated with genes responsible for susceptibility to complex human diseases with a clinically available sample size is still a major challenge for whole genome association study. In addition to including a large number of SNPs, the chance of detecting significant association also requires a very large number of samples owing to the low phenotypic effect of the genes involved in multifactorial diseases. Although, high-throughput genotyping techniques are readily available for handling large sample size and a large number of genetic markers, the cost is still very high. Therefore, methods for estimation of SNP allele frequencies in such studies should be amenable to scaling-up both in the number of loci and in the number of samples. DNA pooling is a well established method, which can vastly reduce the amounts of effort, labor and cost involved in large-scale association studies [26]. Pooling allows one to estimate the allele frequency among large numbers of individuals by examining a single or much fewer samples, reducing the workload from hundreds of samples to one or very a few. Instead of genotyping the large numbers of SNPs in individual samples on Affymetrix SNP chip for genome-wide association scans, a large number of papers have addressed pooling the DNA from large numbers of individuals [27-30].

In the present study, we report a new microarray-based strategy to estimate the allele frequencies of pooled samples. The method is highly sensitive and can be used to analyze a large number of markers and multi-pools simultaneously. Association studies with multifactorial subdivision strategy provide a powerful tool for the study of complex diseases and quantitative traits, influenced by disease heterogeneity, gene-gene and gene-environment interactions. Our method meets the need for the association studies using pooling strategy more elaborate than the two-pool (case vs. control) design. The populations of cases and controls can be stratified on the basis of secondary and tertiary traits to construct a series of sub-pools. In addition to some traits, factors such as age at onset, sex, lifestyle, or other clinical descriptions can also be used for categorization. This might capture effects of environmental factors that are known to affect the disease trait in question. The association studies with multifactorial subdivision strategy provide a powerful tool for the study of complex common disease and quantitative traits, influenced by the effects of disease heterogeneity, gene-gene and gene-environment interactions.

Genotyping pooled samples based on subdivision using microarray significantly reduces workload and cost with similar statistical power compared with genotyping individual samples. Assuming 1000 case and 1000 control samples with 100 candidate SNPs for a disease are to be studied and 100 samples in each pool, using our method, the 2000 PCR products can be analyzed by a single microarray, and a single hybridization reaction. Its statistical power is equivalent to 2 × 10^5 ^individual genotyping (100 SNPs × 1000 cases + 100 SNPs × 1000 controls), which is over a 100-fold reduction. In addition, we introduce the strategy of employing two universal florescence-labeled (Cy3 and Cy5) tags to form the sandwich structure with SNP detection probes and allelic sequences. Regardless the number of SNPs that need to be analyzed, only two universal florescence-labeled tags are needed. Therefore, it vastly reduces the experiment cost.

The fluorescent intensities of the two colors on a microarray spot are not directly proportional to the allele frequencies in pooled DNA samples. Differences in various aspects of oligonucleotide hybridization make it difficult to estimate allele frequencies based on the fluorescence signals. We have tackled this problem by theoretically deducing a Langmuir-type formula for each allele. This Langmuir-type isotherm model integrates the thermodynamics of immobilization and hybridization processes on microarray surface, and describes the relationship between the fluorescence intensity and allele concentration in the spotting solution. Biased amplification of different alleles may occur when the size range of microsatellite alleles is sufficiently great. However, no significant biased amplification with SNP alleles (only one-base difference which is not size difference) was observed in our experiments. Therefore, allele frequencies in the spotting solution can be directly considered as allele frequencies in pooled DNA.

Our method has advantages over other published protocols in its high sensitivity and capability to detect minor difference between allele frequencies. Compared to determination of allele frequencies in individual pools, it is more important to learn whether the allele frequencies among pools and sub-pools are significantly different from each other. The experimental results demonstrate that our method can successfully distinguish allele frequencies differing by 0.01 in the actual pool of clinical samples (*P *< 0.0078). Our results also demonstrate that alleles with a frequency as low as 2% can be detected. Our approach exemplifies the reproducibility of measurement with the mean divergence between individual and pooled allele frequencies of 0.7%, ranging from 0.05% to 2.2%. The observed SEMs varied from 0.0044 to 0.0103.

## Conclusion

In conclusion, we have developed an accurate, robust and high-throughput method to estimate the allele frequency in a large number of samples by pooled DNA samples followed by PCR amplification and microarray analysis. The dynamics of the immobilization and hybridization of the PCR products on the solid surface was studied. The kinetics of these two processes was integrated to estimate the allele frequency in pooled DNA by establishing a Langmuir-type kinetic model. Our approach is inexpensive, efficient and capable of detecting interesting polymorphic differences in candidate gene association studies and genome-wide linkage disequilibrium scans.

## Methods

### DNA stocks, oligonucleotides and other reagents

The synthetic DNA stocks for SNPs *ESR1E-U11 (*T/C) *ESR1F-U21 *(A/G) and, *TGFB1D-U2 *(G/C), primers, probes and fluorescence-labeled tags were purchased from Songon Inc. (Shanghai, China), purified by reverse-phase HPLC using a standard procedure, and dried in vacuo. The plasmids containing inserts with the three point mutations (*HBB17(A/T), HBB28(T/C), HBB26(C/T)*) were constructed using a site-directed mutation technology. The genomic DNAs with the *HBB28(T/C) *mutation were from thalassemia patients, and provided by the First Affiliated Hospital of Gongxi Medical University. All other chemicals and solvents were purchased from Sigma and Gibco BRL (Carlsbad, CA, USA), and used without additional purification. Unless otherwise noted, all samples and buffers were prepared in deionized water prepared using a Milli-Q water purification system (Millipore Corp., Bedford, MA, USA).

### Preparation of pooled DNA samples

DNA samples including the synthetic DNA stocks and plasmids were homozygous for either one or the other allele of the SNPs. The pooled DNA samples for one, SNP (*HBB28*), were prepared from the genomic DNA of 100 patients and normal individuals. These genomic DNA samples were isolated from lymphocytes in peripheral blood of clinical patients using Trizol reagent and dissolved in TE buffer. In all experiments, all the synthetic DNA stocks, plasmids and genomic DNA samples, were initially diluted to a concentration of 30 ng/μl and then mixed gently and requantitated to a working concentration of 10 ng/μl (± 0.1 ng/μl) using NanoDrop ND-1000 Spectrophotometer (NanoDrop Technologies, DE, USA). Pooled DNA samples were prepares based on their genotype for each given SNP.

### PCR amplification

Pooled DNA samples were amplified by using the TaKaRa PCR kits (rTaq) (Kyoto, Japan) with a thermal cycler (PTC-225, MJ Research, Waltham, MA, USA). PCR samples were initially heated up to 96°C for 3 min, and then amplified for 35 cycles. Each thermal cycle consisted of 30s at 94°C, 30s at 55°C, and 30s at 72°C. A final extension step was included for 5 min at 72°C. The sequences of PCR primers for amplification of the polymorphic sequences containing SNPs were TTCATCTGAGTTCCAAATGTCC and AATATACAATTATTTCAGAACCATTAGAGAC for *ESR1E-U11*; AGCTGTTTTATGCTTTGTCTCTG and AGGAATATACAATTATTTCAGAACCATT for *ESR1F-U21*; TGCTGCYGCTGCTGCTAC and CTCCATGTCGATAGTCTTGCA for *TGFB1D-U2*; AGGGTTGGCCAATCTACTCC and GTCTCCACATGCCCAGTTTC for *HBB17, HBB28 *and *HBB26*. PCR products were analyzed by gel electrophoresis with 1% agarose, and purified by precipitation with sodium acetate and 100% ice-cold alcohol, then washed in 75% alcohol several times and finally dried in DNA vacuum.

### Microarrays fabrication of the PCR products

All PCR products representing different allele ratios were dissolved in a printing buffer (3 × SSC solution), and quantitated to a working concentration of 50 μM. PCR products were spotted into five identical matrixes on an aldehyde-coated slide (CEL Associates, Pearland, TX, USA). Within each matrix PCR products for synthetic DNA and plasmid samples were spotted in quintuplicate, and those for genomics DNA samples were spotted twenty times. Spots were ~200 μm in diameter and ~300 μm between adjacent centers. Printing was performed using the contact printing robot (SpotBot, Telechem International, CA, USA). After printing, all printed slides were hydrated overnight at 37°C in a box containing 200 ml saturated NaCl solution. After hydration, the slides were exposed to UV light at 950 mJ/cm^2 ^in Stratalinker 2400 (Stratagene, CA, USA), then rinsed in boiling water for 2 min to denature the PCR product followed by rinsing in 100% alcohol for 1 min, dried by centrifugation at 1000 r.p.m. for 1 min, and stored in a vacuum oven [31].

### Hybridization and microarray scan

Two hybridization processes were involved in microarray analysis: hybridization of the tags to the probes, and hybridization of the tag-probe duplexes to the allelic sequences. All SNP probes, which would hybridize with the corresponding DNA pool samples of the studied SNP markers in a matrix, were dissolved in 2 × hybridization buffer (Agilent Technologies. Inc. USA), and mixed with equal volume of Cy3/Cy5 labeled tags solution at a molar ratio of 1:2 to react for 30 min. Then the mixture was added to the hybridization matrix in the glass. The microarray was incubated overnight at 42°C in a hybridization cassette (Telechem International). After hybridization, the slide was sequentially rinsed in 2 × SSC/0.1% SDS and in 2 × SSC at 37°C, 5 min for each step, and dried by centrifugation. The slides were then scanned using a Genepix 4000B scanner (Axon Instruments, Foster City, CA, USA) at a resolution of 5 μm with 100% excitation intensity with PMT set to 55% and 48% for Cy5 and Cy3 channels, respectively. Spot analysis and quantification of the original 16-bit TIF images were performed with the Genepix software (v 5.0).

### Statistical analysis

Standard deviation (SD) was calculated for the signal intensities of the repeats of each pooled sample. The significance levels of the differences between the allele frequencies for the all comparison were analyzed by the Kolmogorov-Smirnov test. The *P *value was used as a measurement of the degree of significance between the measured and known allele frequencies. The statistical software Systat 12.0 (Systat Software Inc., San Jose, CA, USA) was used to perform the data analyses.

## Abbreviations

SNPs: Single nucleotide polymorphisms; BAMPER: Bioluminometric assay coupled with modified primer extension reactions; RFLP: Restriction fragment length polymorphism; DASH: Dynamic allele-specific hybridization; SSCP: Single strand conformation polymorphism; ARMS: Amplification refractory mutation system; HPLC: High performance liquid chromatography.

Human genes: *ESR1*: estrogen receptor 1, Genbank No. NM_000125.2, OMIM#133430; *TGFB1*: transforming growth factor, Genbank No. NM_000660.3, OMIM#190180; *HBB*: hemoglobin, beta, Genbank No. NM_000518.4, OMIM#141900.

## Authors' contributions

BCYe and BCYin conceived and designed the study and manuscript preparation. BCYe oversaw the project. HL contributed to discussions and manuscript preparation.
